# A comprehensive survival and prognosis analysis of GPR55 expression in hepatocellular carcinoma

**DOI:** 10.18632/aging.205008

**Published:** 2023-09-08

**Authors:** Tianyu Wang, Kang Xia, Tao Qiu, Shangting Han, Zhongbao Chen, Xiaoxiong Ma, Long Zhang, Jilin Zou, Yalong Zhang, Bo Yu, Chenyang Kong, Jiayu Guo, Yiting Liu, Jiangqiao Zhou, Shusen Zheng

**Affiliations:** 1 Department of Organ Transplantation, Renmin Hospital of Wuhan University, Wuhan, China; 2 Department of Urology, Renmin Hospital of Wuhan University, Wuhan, China; 3 Department of General Surgery, Renmin Hospital of Wuhan University, Wuhan, China; 4 Department of Surgery, Division of Hepatobiliary and Pancreatic Surgery, The First Affiliated Hospital, Zhejiang University School of Medicine, Hangzhou, China; 5 NHC Key Laboratory of Combined Multi-organ Transplantation, Hangzhou, China; 6 Key Laboratory of the Diagnosis and Treatment of Organ Transplantation, Research Unit of Collaborative Diagnosis and Treatment for Hepatobiliary and Pancreatic Cancer, Chinese Academy of Medical Sciences (2019RU019), Hangzhou, China; 7 Key Laboratory of Organ Transplantation, Research Center for Diagnosis and Treatment of Hepatobiliary Diseases, Hangzhou 310003, Zhejiang, China

**Keywords:** survival and prognosis analysis, GPR55, hepatocellular carcinoma, mitogen-activated protein kinase signaling pathway

## Abstract

Hepatocellular carcinoma (HCC) is the most common subtype, accounting for about 90% of all primary liver cancers. The liver is rich in a large number of immune cells, thus forming a special immune microenvironment, which plays a key role in the occurrence and development of hepatocellular carcinoma. Nowadays, tumor immunotherapy has become one of the most promising cancer treatment methods. Immune checkpoint inhibitors (ICIs) combined with VEGF inhibitors are listed as first-line treatment options for advanced HCC. Therefore, the search for a potential biomarker to predict the response to immunotherapy in HCC patients is urgently needed. The G protein-coupled receptor 55 (*GPR55*), a lysophosphatidylinositol (LPI) receptor, has recently emerged as a potential new target for anti-tumor therapy. Previous studies have found that *GPR55* is highly expressed in breast cancer, pancreatic cancer, skin cancer and cholangiocarcinoma, and is involved in tumor proliferation and migration. However, the role and mechanism of *GPR55* in HCC has not been elucidated. Therefore, this article discusses the clinical significance of *GPR55* in HCC and its correlation with the immune response of HCC patients, so as to provide theoretical basis for improving the prognosis of HCC.

## INTRODUCTION

Liver cancer, including hepatocellular carcinoma (HCC) and intrahepatic cholangiocarcinoma, is the fifth most common malignant neoplasm in the United States [1]. Most HCC patients are diagnosed when already in an advanced stage due to the potential difficulties in their early detection [2]. As advanced HCC is highly malignant with rapid progression, treatment for these patients is difficult and the therapeutic effect is generally unsatisfactory, leading to undesirable survival outcomes. Due to the promising efficacy in patients with advanced tumor-stage melanoma or lung cancer, immunotherapy represented by immune checkpoint inhibitors has quickly entered clinical trials for the treatment of patients with advanced HCC [3, 4]. Regulation of the expression of checkpoint-related genes to improve the tumor microenvironment in HCC may restore immune recognition and immunogenicity, and strengthen the response to immunotherapy [5]. Therefore, the identification of a promising biomarker for the prediction of immunotherapy response and prognosis in patients with HCC and the elucidating its potential role in the immune microenvironment of HCC is quite significant.

G-protein-coupled receptors (GPCRs) are one of the largest families of membrane receptors and exert various cellular pathways through the activation of G-proteins [6]. The GPCR 55 (*GPR55*), which belongs to the GPCR family, is a seven transmembrane receptor with wide expression in the brain and peripheral tissues [7]. A recent study highlighted that *GPR55* plays a pro-inflammatory role in the immune response, indicating that *GPR55* could be a new immune therapeutic target [8]. The regulatory role of *GPR55* in HCC is mainly located in immune cells, because the liver is rich in a large number of immune active cells, including Kupffer cells, dendritic cells, liver endothelial cells, etc. The tumor microenvironment of HCC also contains complex immune cell population, tumor cells and cytokine environment [9]. However, no studies have evaluated the clinical impact of *GPR55* expression in HCC. In this present study, our objective was to provide more information on the clinical importance of *GPR55* and its correlation with the immune response in patients with HCC.

## METHODS

### Comprehensive bioinformatics analysis

We downloaded original sequencing data from the HCC datasets from the GEO database. The UCSC Xena database (http://xena.ucsc.edu/) was used to retrieved the original gene expression and methylation data from the LIHC dataset. The survival importance of *GPR55* in HCC was evaluated using the Kaplan-Meier Plotter database (http://kmplot.com/analysis/index.php?p=background). ImmuCellAI (http://bioinfo.life.hust.edu.cn/ImmuCellAI#!/) and TIMER (https://cistrome.shinyapps.io/timer/) databases were searched to define the correlation between *GPR55* expression and infiltration of immune cell types in HCC specimens. T-cell inflammation (TIS), defined by ImmuCellAI, is an objective indicator to predict the response to immunotherapy in HCC. The single-sample gene set enrichment analysis (ssGSEA, GSE22058, GSE46444, GSE63898, GSE76427, GSE25097) was conducted using the R package R ‘GSVA’ to investigate the relationship between *GPR55* expression and 28 types of immune cells in HCC. We also used the ‘GSVA’ package to determine the relationship between *GPR55* expression and immune status represented by 25 sets of immune-related genes.

### Enrichment analysis

Patients in The Cancer Genome Atlas (TCGA)-LIHC data set were divided into subgroups with high *GPR55* expression and low *GPR55* expression according to the median *GPR55* gene transcription. Differentially expressed genes (DEG) of the two subgroups were determined by the R package ‘limma’. The Gene Ontology Database (GO), Molecular Function (MF), Cellular Component (CC), and Kyoto Encyclopedia of Genes and Genomes (KEGG) functional analyses were used to annotate the Gene set with the R package ‘clusterProfiler’.

### Cell culture and treatment

The HCC HepG2 and 97H cell lines used in this study were obtained from the Chinese Academy of Sciences Type Culture Collection of the Chinese Academy of Sciences (Shanghai, China) and were maintained in Dulbecco’s modified Eagle’s medium (Gibco, Carlsbad, CA, USA) supplemented with 10% fetal bovine serum (FBS; Gibco) and 0.1% penicillin-streptomycin solution (Gibco). Cells were incubated at 37°C in a humidified atmosphere with 5% CO_2_.

### Plasmids, adenovirus, and stable cell line construction

*GPR55* shRNA and overexpression plasmids were constructed with the indicated vectors. *GPR55* shRNA and overexpressed lentiviruses were packaged in HEK293 cells using a double-packing plasmid system. Lentiviruses were collected and added to HepG2 and 97H cells to obtain stable cell lines.

### Cell-Counting Kit-8 assay

Cell viability was examined using a Cell-Counting Kit-8 (CCK-8) following the manufacturer’s protocols (C0037, Beyotime). HepG2 cells and 97H cells were inoculated in 96-well plates (AB3396, Thermo Fisher Scientific). After cells had adhered to the plate, they were further cultured for 0, 24, 48, 72, and 96 h, respectively. CCK8 reagents (10 μL) were added and the absorbance at 450 nm was measured.

### Cell migration assay

Healthy HepG2 cells and 97H cells were resuspended in DMEM. Then they were seeded at 3 × 10^4^ cells/well in the upper compartment of a Transwell™ chamber (72401, NEST). Meanwhile, DMEM containing 600 μL of 2% FBS or 600 μL of 10% FBS was added to the lower chamber, respectively. Cells were cultured for 6 h or 24 h (HepG2) or 6 h or 48 h (97H) with phosphate buffered saline. Then, 600 μL of 4% paraformaldehyde solution was used to fix cells for 15 min at room temperature and 600 μL of 0.1% crystal violet (C0121, Beyotime) was used to stain cells for 1 h at 37°C. The images were obtained under a positive microscope. The number of positively stained cells reflected the cell-migration ability.

### Immunofluorescence

*GPR55* (DF2752, Affinity), PD19 (#86163, CST), PD-L1 (#13684, CST) antibody immunofluorescence staining was used to detect the expression of liver cancer tissue. After primary antibody was incubated at 4°C overnight, liver cancer sections were incubated with secondary antibody. The nuclei are labeled with DAPI. Immunofluorescence images were captured and analyzed using Image-Pro Plus (version 6.0). HCC tissue samples were collected from Renmin Hospital of Wuhan University (Wuhan, P. R. China). This study obtained the informed consent of all patients and the approval of Ethics Review Board of Renmin Hospital of Wuhan University.

### Western blotting

Proteins were extracted from hepatoma cell lines (HepG2 and 97H) using standard procedures. Protein concentrations were measured using a BCA protein assay kit (Thermo Fisher Scientific, 23227). A 10% SDS-PAGE gel was prepared and protein samples of the same mass were added to the loading buffer for separation. After sealing membranes in 5% skim milk powder at room temperature for 60 min, the primary antibody was added and incubated at 4°C overnight. Secondary antibodies of the respective species were added, incubated at room temperature for 60 min, rinsed 3 times with TBS-T buffer, and signals were developed using an enhanced chemiluminescent substrate (Thermo Fisher Scientific, A38556), and captured using a gel imaging system (ChemiDoc XRS +). ImageJ software was used to quantify protein expression levels.

### Statistical analysis

The continuous data was expressed as Mean ± SD and categorical variables were presented as counts and percentages. All statistical analysis of the above data was completed with SPSS version 20.0 and with R version 3.0. The correlation between the expression of *GPR55* mRNA and clinical characteristics in individuals with HCC was measured using the Chi-square test. The survival significance associated with *GPR55* mRNA expression was determined by Kaplan-Meier curves and statistically evaluated by the logarithmic rank test. The correlation between *GPR55* and immune cells, and expression of immune checkpoints was shown by correlation curves and statistically evaluated by Spearman’s correlation analysis. A *P*-value of <0.05 was considered statistically significant.

## RESULTS

### *GPR55* expression patterns in HCC

**Figure 1:**
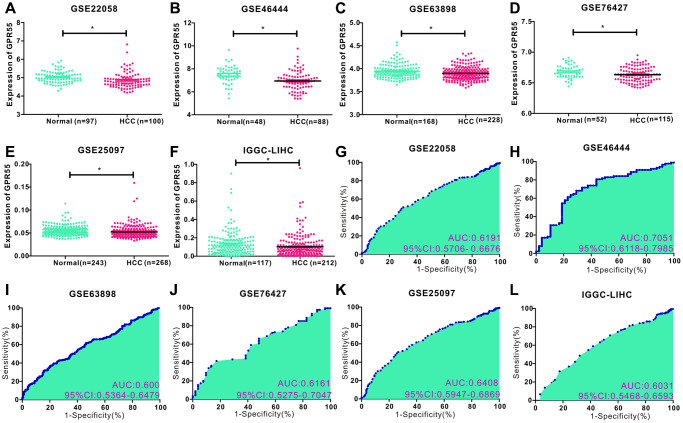
(**A**–**F**) Comparison of GPR55 expression in normal and tumor tissues in different HCC data sets (GSE22058, GSE46444, GSE63898, GSE76427, GSE25097, IGGC-LIHC) (^*^P < 0.05). (**G–L**) ROC diagnostic curve of GPR55 in different data sets.

The pattern of *GPR55* expression was explored in different HCC datasets. We downloaded the original sequencing data of HCC from five GEO datasets (Figure 1A–1F), and the comparison graphs revealed that *GPR55* mRNA expression was downregulated in HCC tissues compared to its expression in correspondingly normal liver tissues. In addition, we also validated this in the TCGA-LIHC dataset (Supplementary Figure 1A). Subsequently, we investigated whether the expression of *GPR55* could serve as a diagnostic index to differentiate HCC from normal liver tissues. The ROC curves demonstrated that *GPR55* mRNA displayed good diagnostic value for differentiating HCC from liver tissues (Figure 1G–1L). However, its ROC value was only 0.533 in the TCGA-LIHC database (Supplementary Figure 1B). Hence, we could observe that the expression of *GPR55* was downregulated in HCC tissues (*N* = 1011) compared with normal liver tissues (*N* = 725), and it could act as a diagnostic marker for HCC.

### Clinical significance of *GPR55* expression in HCC

**Figure 2:**
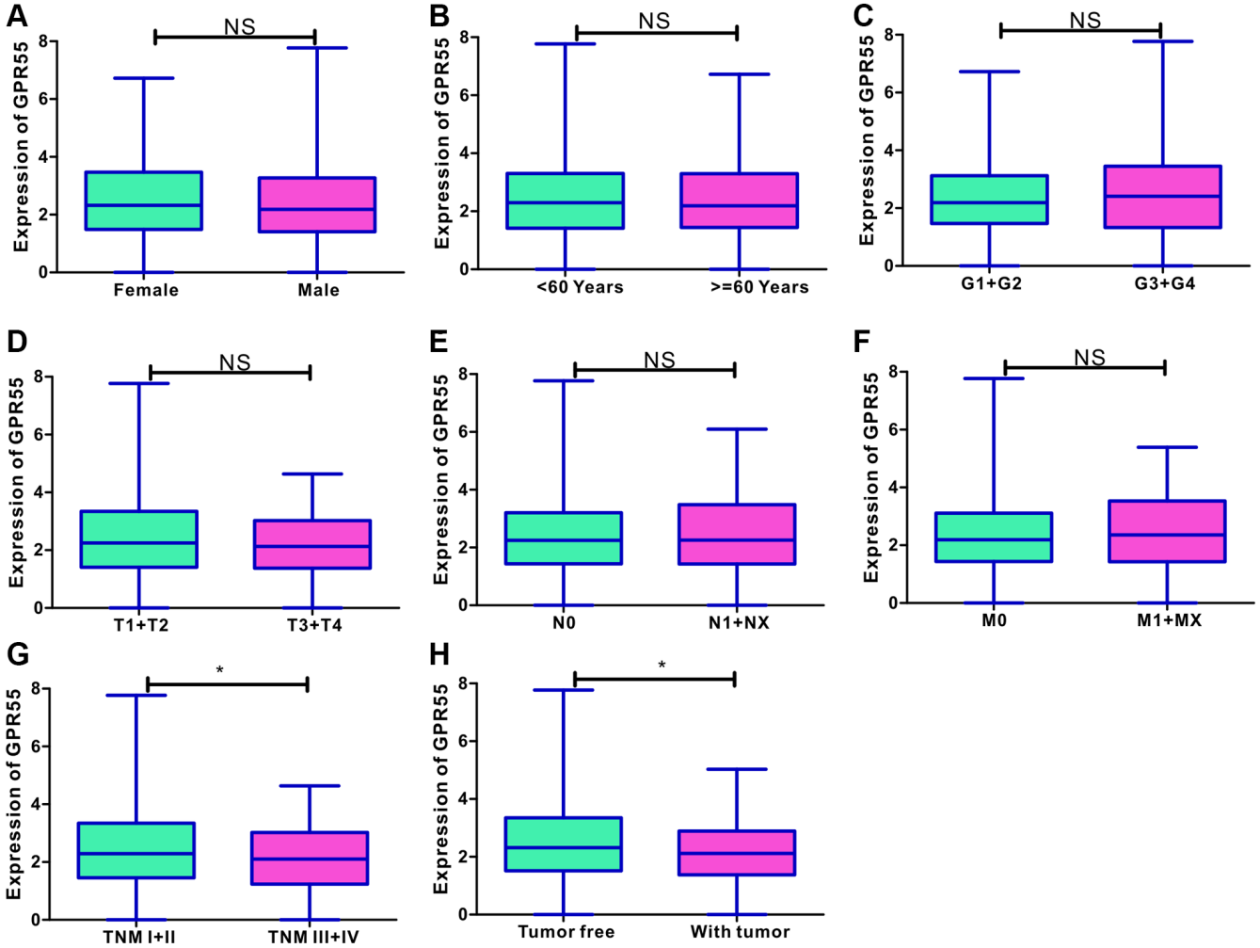
(**A**–**F**) Correlation analysis of GPR55 with age, sex, histological grade, TNM stage and tumor status. (**G**, **H**) The dot plots of GPR55 expression in HCC individuals with TNM I/II stage, and in tumor free individuals. (^*^P < 0.05).

To clarify the clinical importance of *GPR55* expression in HCC, we specifically analyzed the correlation between *GPR55* expression and clinical parameters based on TCGA-LIHC data set. We analyzed its correlation with age, sex, histological grade, TNM stage, and tumor status (Figure 2A–2F), and the dot plots showed that the expression of *GPR55* mRNA was higher in HCC individuals with TNM I/II stage, and in tumor-free individuals (Figure 2G, 2H). In summary, *GPR55* mRNA expression was associated with better clinical features of HCC.

### Kaplan-Meier curves of *GPR55* in individuals with HCC

**Figure 3:**
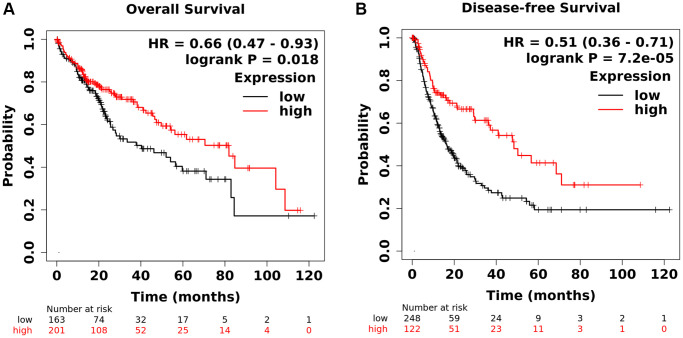
(**A**) Analysis of total survival of GPR55. (**B**) Analysis of disease-free survival of GPR55.

To gain a better understanding of the effects of abnormal *GPR55* expression on survival outcomes, we performed a pancancer analysis using Kaplan-Meier plotter. As shown in Supplementary Table 1, the survival analysis revealed that *GPR55* was associated with different prognostic significance for different malignant neoplasms. Pancancer analysis revealed that overexpression of *GPR55* is a protective factor for much better overall survival and better disease-free survival in HCC, breast cancer, and Uterine Corpus Endometrioid Carcinoma. However, overexpression of *GPR55* was a protective biomarker for better overall survival and a risk factor for disease-free survival in HNSC. The survival curves of *GPR55* in individuals with HCC are presented in Figure 3, which confirmed our observation that upregulation of *GPR55* was not only correlated with better overall survival (Figure 3A), but also associated with better disease-free survival (Figure 3B). We also performed subgroup analyzes based on clinical metrics among individuals with HCC; detailed results are shown in Table 1.

**Table 1:** Subgroup analysis of clinical indicators of HCC individuals.

**Clinical Feature**		**HR**	**95% CI**	***P* value**	**HR**	**95% CI**	***P* value**
Gender	Female	0.35	0.2–0.65	0.00018	0.46	0.27–0.76	0.0022
	Male	0.73	0.44–1.22	0.23	0.49	0.32–0.75	7*e-4
Race	White	0.55	0.35–0.87	0.0089	0.62	0.4–0.96	0.031
	Asian	0.77	0.42–1.38	0.37	0.46	0.26–0.81	0.0059
Alcohol consumption	Yes	1.71	0.77–3.84	0.19	0.58	0.34–0.97	0.036
	No	0.6	0.38–0.94	0.025	0.36	0.22–0.61	6*e-5
Hepatitis virus	Yes	0.68	0.34–1.34	0.26	0.33	0.19–0.58	5*e-5
	No	0.59	0.37–0.92	0.019	0.6	0.37–0.98	0.041
Vascular invasion	None	0.58	0.35–0.97	0.037	0.45	0.27–0.74	0.0013
	Micro	1.63	0.65–4.06	0.29	0.52	0.27–1.0	0.048
Grade	1	0.57	0.22–1.45	0.23	0.55	0.21–1.47	0.23
	2	0.59	0.35–1	0.047	0.46	0.28–0.76	0.002
	3	0.64	0.34–1.18	0.15	0.42	0.25–0.71	0.00053
T stage	T1	0.59	0.32–1.07	0.078	0.57	0.35–0.94	0.024
	T2	0.51	0.23–1.1	0.082	0.29	0.13–0.62	0.00088
	T3	1.59	0.86–2.95	0.14	0.62	0.34–1.12	0.11
TNM stage	I	0.61	0.32–1.15	0.12	0.57	0.33–0.98	0.0389
	II	0.6	0.27–1.35	0.21	0.3	0.14–0.65	0.0015
	III	1.57	0.84–2.92	0.16	0.57	0.31–1.02	0.055
Sorafenib	Treated	0.43	0.12–1.54	0.18	0.49	0.21–1.12	0.085

**Figure 4:**
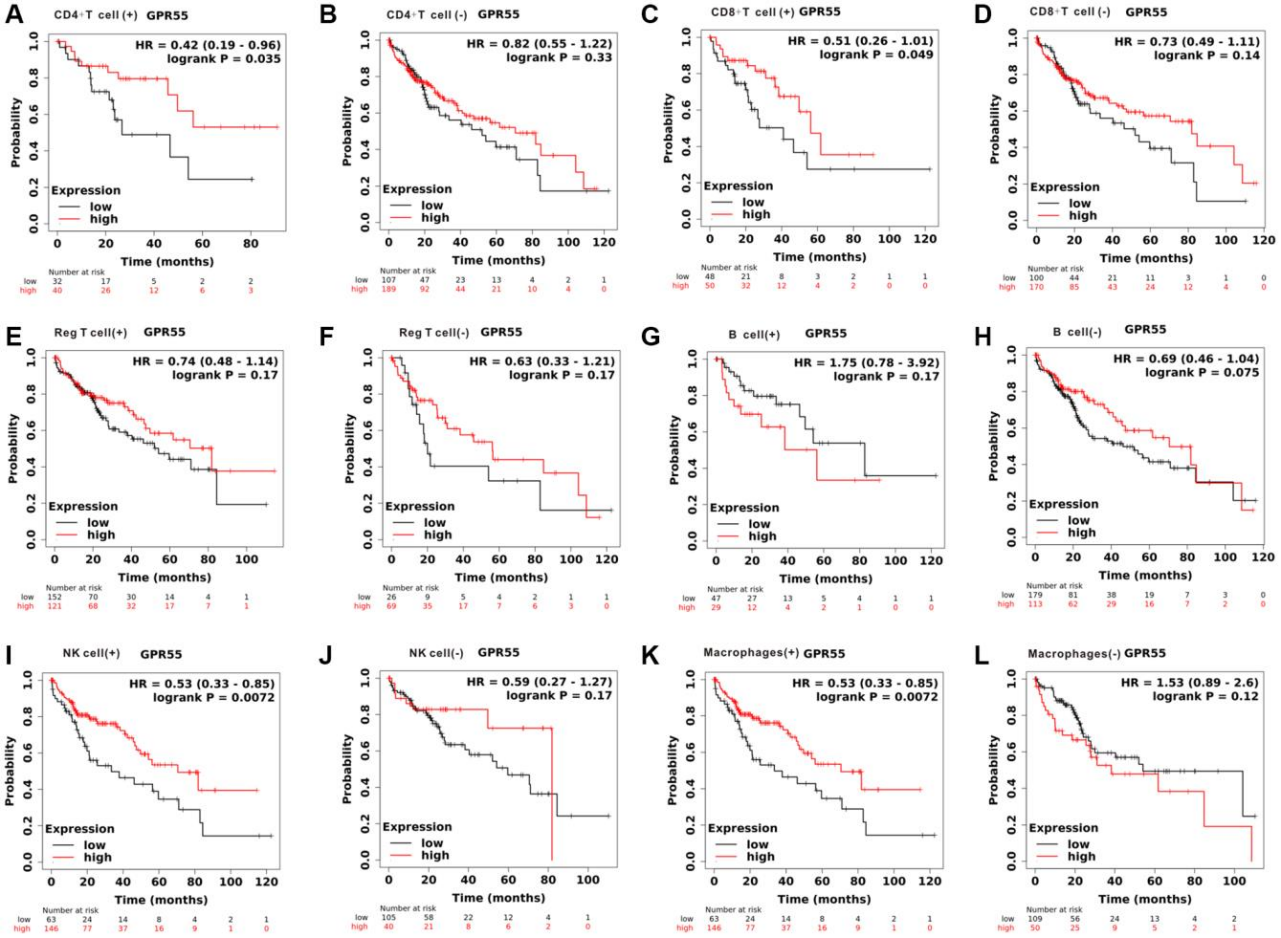
**Total survival analysis of GPR55 expression in HCC with different immune cell subsets.** (**A**, **B**) CD4^+^T cell. (**C**, **D**) CD8^+^T cell. (**E**, **F**) Reg T cell. (**G**, **H**) B cell. (**I**, **J**) NK cell. (**K**, **L**) Macrophages.

**Figure 5:**
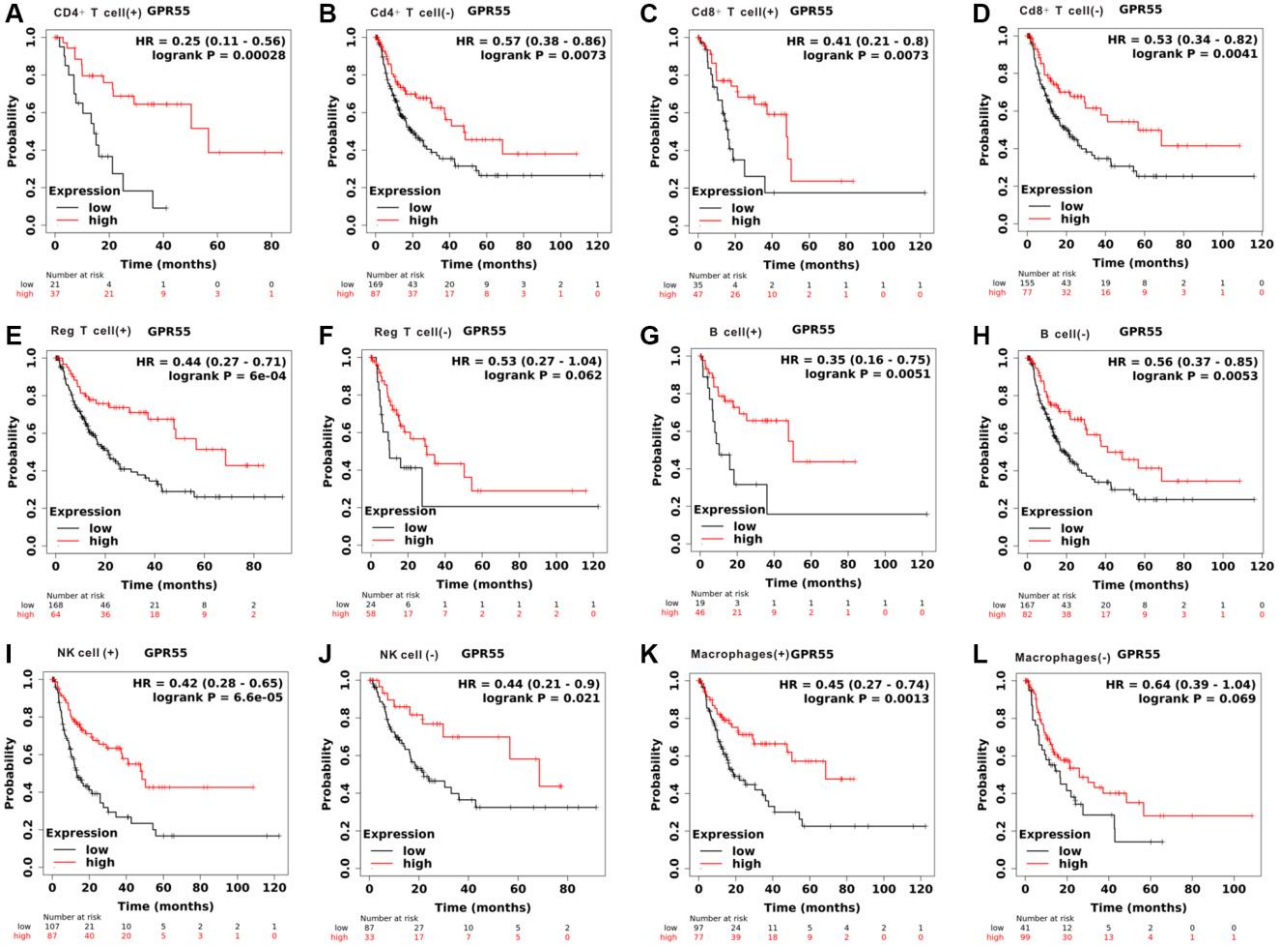
**Disease-free survival analysis of GPR55 expression in HCC with different immune cell subsets.** (**A**, **B**) CD4^+^T cell. (**C**, **D**) CD8^+^T cell. (**E**, **F**) Reg T cell. (**G**, **H**) B cell. (**I**, **J**) NK cell. (**K**, **L**) Macrophages.

Due to the close relationship between *GPR55* expression and prognosis in HCC, we further explored whether *GPR55* expression influenced HCC survival outcomes due to immune infiltration. We conducted a prognostic analyzes based on *GPR55* expression in HCC in different subgroups of immune cells. As listed in Figure 4, there was a statistically significant relationship between *GPR55* expression and overall survival of HCC in patients with enriched infiltration of CD4+ cells, CD8+ cells, NK cells, and macrophages, while there was no association in patients with decreased infiltration of immune cells. Regarding relapse-free survival, the prognostic significance was more significant in patients with HCC having enriched immune cell infiltration than in those with decreased immune cell infiltration (Figure 5).

### *GPR55* was associated with immune infiltration in HCC

**Figure 6:**
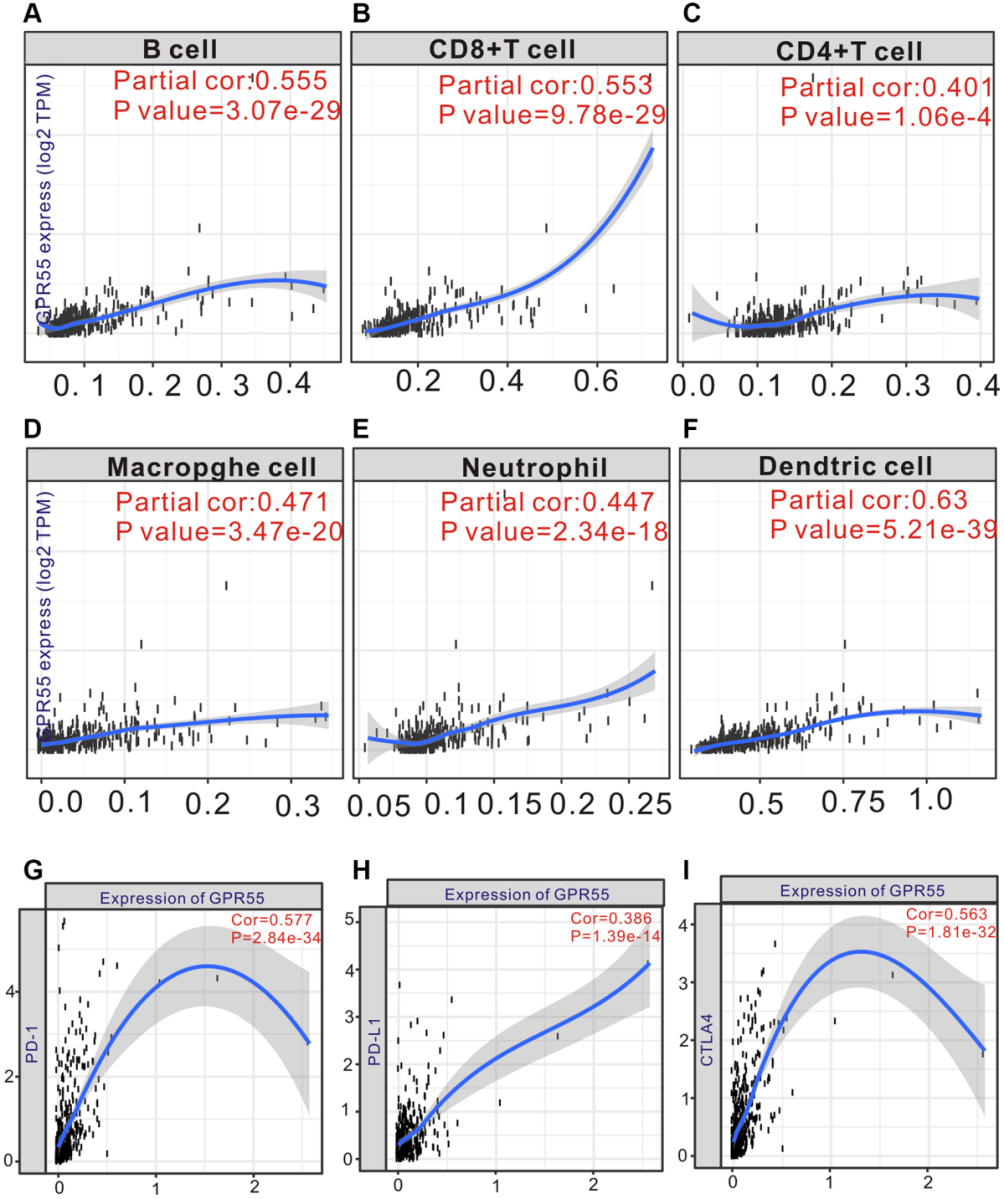
(**A**–**F**) TIMER analysis of GPR55 expression and different infiltrating immune cells in HCC tissue. (**G**–**I**) TIMER analysis of GPR55 expression and different immunotherapy in HCC tissue.

**Figure 7:**
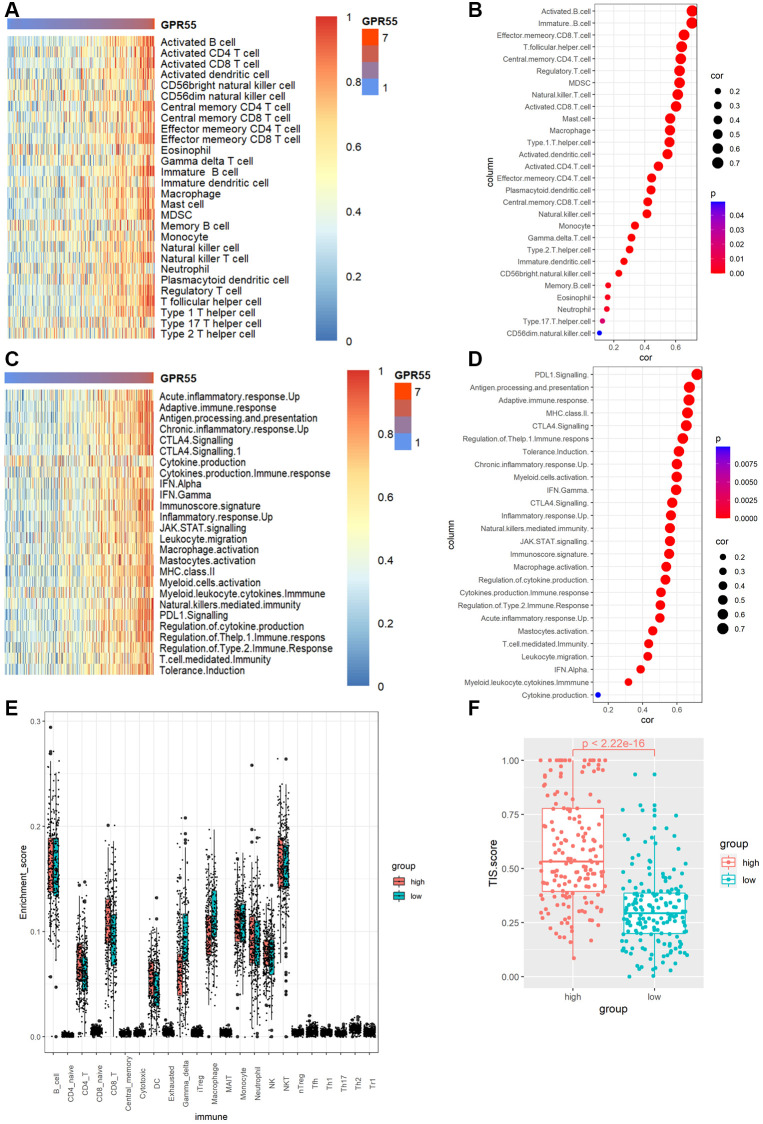
(**A**) Heat map of GPR55 expression in various immune cells. (**B**) Bubble diagram of correlation between GPR55 and various immune cells. (**C**) Heat map of GPR55 expression in various immune cell reactions. (**D**) Bubble diagram of correlation analysis between GPR55 and various immune cell reactions. (**E**) Enrichment fraction of GPR55 high and low expression subgroups in immune cells. (**F**) Analysis of TIS scores in high and low expression groups of GPR55.

As mentioned above, the abundance of immune cells could affect the survival outcomes of patients with HCC; thus, we calculated the relationship of *GPR55* expression with a panel of immune cells infiltrating HCC tissues using the TIMER algorithm. Figure 6A–6F shows that *GPR55* levels exhibited an obviously positive association with B cells (r = 0.555, Pw3.07e^**−**29^), CD8+ T cells (r = 0.553, *P* = 9.78e^−29^), CD4+ T cells (r = 0.401, *P* = 1.06e^−4^), macrophages (r = 0.471, *P* = 3.47e^−20^), neutrophils (r = 0.447, *P* = 2.34e^−18^), and dendritic cells (r = 0.63, *P* = 5.21e^−39^). Meanwhile, the expression of *GPR55* is closely related to the infiltration of common immune cells and immune checkpoint proteins (PD-1, PD-L1, and CTLA4) in HCC (Figure 6G–6I). Critically, high levels of *GPR55* imply a better response rate to HCC immunotherapy, suggesting that *GPR55* is a good indicator for assessing the tumor immune microenvironment and predicting HCC immunotherapy response. To further explore the potential role of *GPR55* in immune cell infiltration in HCC, we used the TIMER database to perform a correlation analysis between *GPR55* and the sets of immune markers of several immunocytes, such as neutrophils, monocytes, NK, T cells, B cells, macrophages, and dendritic cells. As listed in Supplementary Table 2, *GPR55* levels were strongly related to the infiltration of most immune cells in HCC. Furthermore, we used single cell-sequencing analysis to determine which immune cells specifically expressed *GPR55* mRNA in HCC samples. As shown in Figure 7A, we found that *GPR55* is expressed mainly by immune cells in HCC. In addition, we estimated the relationship between *GPR55* expression and infiltration of 28 immune cell types in primary HCC samples using the ssGSEA algorithm. From the heatmap (Figure 7A, 7B), we observed that *GPR55* expression was significantly correlated with most types of immune cells, especially activated B cells, immature B cells, effector CD8+ T cells, follicular helper T cells, central memory CD4 T cells, and regulatory T cells. Given that *GPR55* is expressed mainly in immune cells in HCC, we also quantified the abundance of tumor-infiltrating immune cells (TIIC) in HCC samples using the ImmuCellAI algorithm. It should be noted that the enrichment scores of most TIICs differed between the high and low subgroups of *GPR55*, which was consistent with the ssGSEA algorithm (Figure 7E). Therefore, together with the results of the TIMER database, we inferred that activation of *GPR55* in combination with infiltration of activated B and T cells led to remodeling of the immune microenvironment in HCC.

### Association with immunotherapy response

To gain insight into the possible association between *GPR55* expression in HCC and an active immune phenotype, we used a panel of genes associated with cancer immune responses to explore the immunophenotype in HCC. In Figure 7C, the immune phenotype of HCC tended to be associated with the red genes indicated in the heatmap with increasing expression of *GPR55*. The results of the quantitative analysis (Figure 7D) revealed a close relationship between *GPR55* expression and PDL1 signaling (r = 0.725, *P* = 0.001), antigen processing and presentation (r = 0.721, *P* = 0.001), adaptive immune response (r = 0.717, *P* = 0.001), MHC class II antigens (r = 0.706, *P* = 0.001), CTLA4 signaling (r = 0.695, *P* = 0.001), regulation of the T help1 immune response (r = 0.682, *P* = 0.001), and tolerance induction (r = 0.676, *P* = 0.001). The TIS is a reliable biomarker for the prediction of immunotherapy response, and we used this index to measure the ability of *GPR55* to predict immunotherapy response in patients with HCC. We found that patients with HCC in high *GPR55* expression subgroups reached higher TIS scores (Figure 7F), indicating that they are correlated with a better response to anti-PDL1 therapy.

### Enrichment analysis

**Figure 8:**
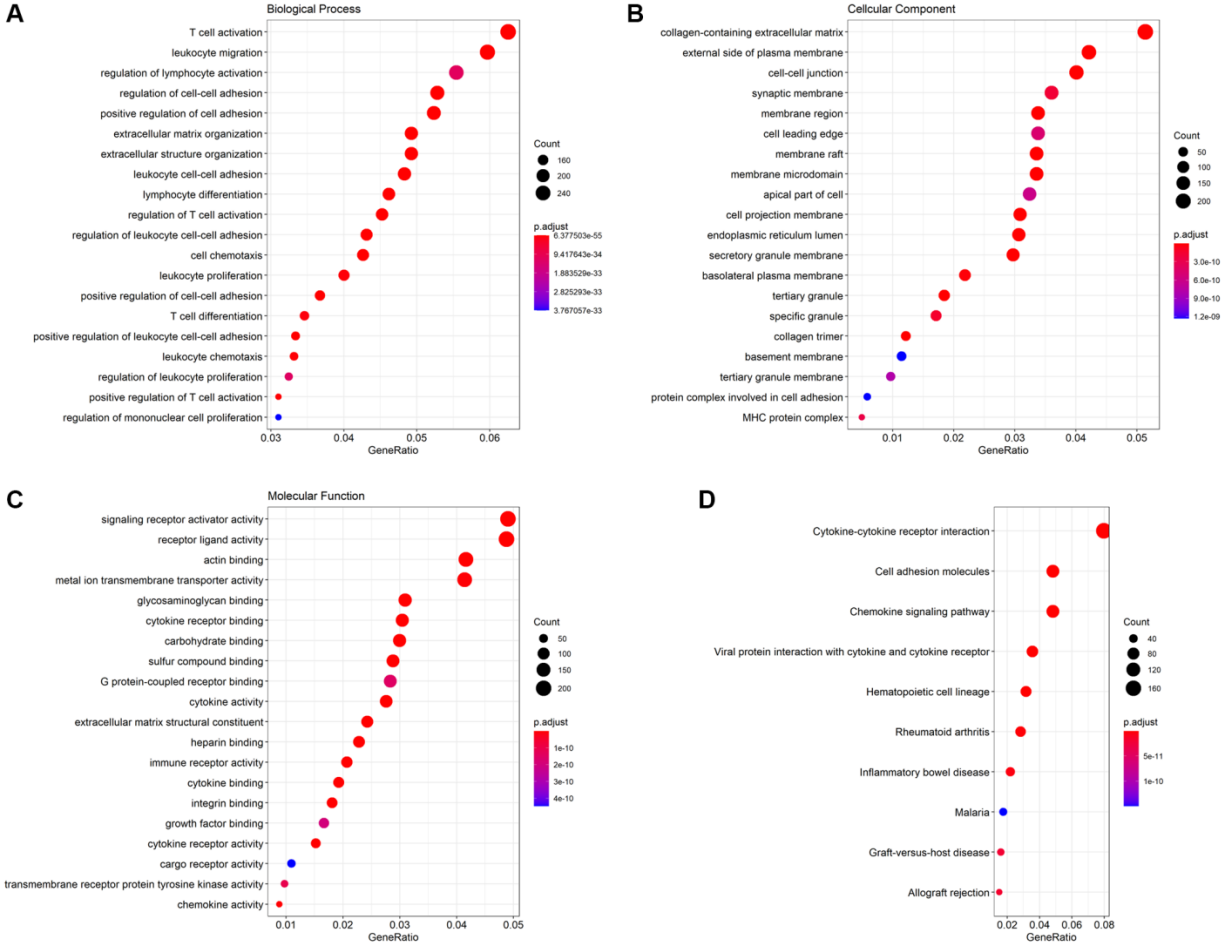
**GO function analysis and KEGG pathway analysis.** (**A**) Biological Process (BP), (**B**) Cellular Component (CC), (**C**) Molecular Function (MF), (**D**) KEGG pathway.

We used the DESeq2 package to identify the downregulated and upregulated genes between *GPR55* high and low expression groups according to the fold-change (FC) value. We subjected the most significant genes (logFC > 2) to enrichment analysis using the Goplot and clusterProfiler packages. The most significant biological processes involving *GPR55* in HCC were T cell activation, leukocyte migration, regulation of cell-cell adhesion, lymphocyte differentiation, and regulation of T cell activation (Figure 8A). The most significant CCs of *GPR55* in HCC were the collagen-containing extracellular matrix, the external side of the plasma membrane, and the cell-cell junction (Figure 8B). The most significant MFs of *GPR55* in HCC were signaling receptor activator activity, receptor ligand activity, and actin binding (Figure 8C). The most significant KEGG pathways involving *GPR55* in HCC were cytokine-cytokine receptor interactions, cell adhesion molecules, and the chemokine signaling pathway (Figure 8D). It can be deduced that *GPR55* is involved mainly in the immune activation in HCC.

### *GPR55* regulated the malignant phenotype of HCC cells

**Figure 9:**
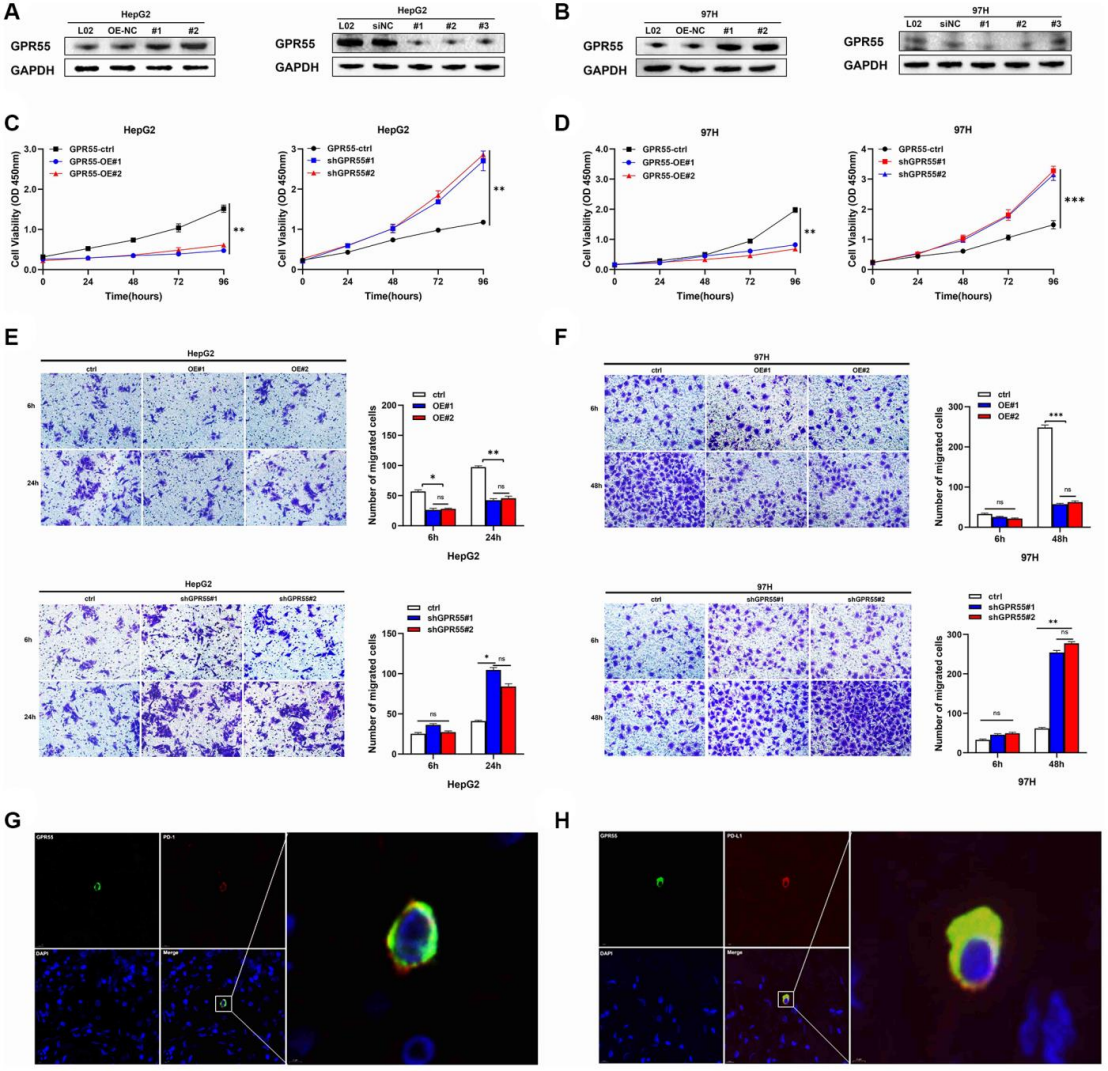
(**A**) Protein levels of GPR55 were detected by western-blotting in HepG2 cells, respectively. (**B**) Protein levels of GPR55 were detected by western-blotting in 97H cells, respectively. (**C**) Determination of cell viability in HepG2 cells. (**D**) Determination of cell viability in 97H cells. (**E**) Transwell assay in HepG2 cells. (**F**) Transwell assay in 97H cells. (**G**) Immunofluorescence staining of GPR55 and PD-1. (**H**) Immunofluorescence staining of GPR55 and PD-L1. *N* = 3, ^***^P < 0.01, *t*-test.

To investigate the regulatory role of GPR55 in the proliferation, migration, and invasion of HCC cells, we examined the expression levels of GPR55 in four HCC cell lines (Supplementary Figure 2). Subsequently, we stably upregulated the expression of GPR55 in HepG2 and 97H cells by lentiviral transfection. As shown in the western blotting bands, compared to the control group, HCC cells with overexpressed *GPR55* showed significantly higher expression of the *GPR55* protein, while exposure to specific short hairpin RNA significantly reduced the expression of *GPR55* (Figure 9A, 9B). Subsequently, we used the CCK-8 assay to assess the effect of *GPR55* on the proliferation capacity of HCC cells. Cell viability in the *GPR55* overexpression group was markedly decreased compared to the control group, and cell viability in the low expression group of *GPR55* was markedly increased compared to the control group (Figure 9C, 9D). Further, the Transwell assay showed that the migration and invasion ability of HepG2 and 97H cells in the *GPR55* overexpression group was weaker than that of the control cells and the *GPR55* low expression group showed the opposite effects (Figure 9E, 9F). Immunofluorescence assay showed that *GPR55* was co-expressed with PD-1 and PD-L1, reflecting the potential of *GPR55* in immunotherapy (Figure 9G, 9H).

### *GPR55* regulated the proliferation and migration of HCC cells through MAPK signaling

**Figure 10:**
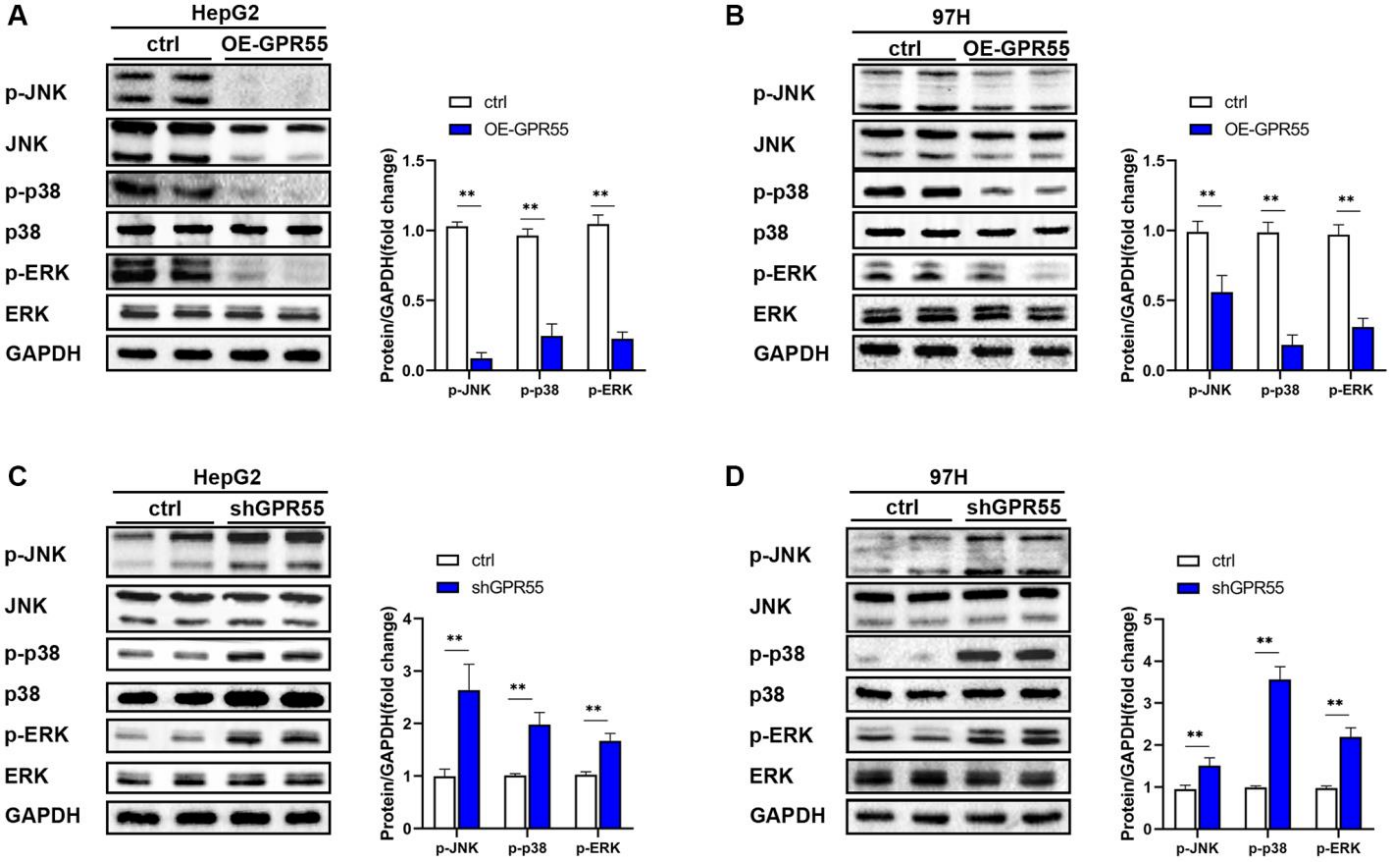
(**A**) The expression of MAPK pathway related proteins in GPR55 overexpression group and control group were detected by western-blotting in HepG2 cells, respectively. (**B**) The expression of MAPK pathway related proteins in GPR55 overexpression group and control group were detected by western-blotting in 97H cells, respectively. (**C**) The expression of MAPK pathway related proteins in GPR55 low expression group and control group were detected by western-blotting in HepG2 cells, respectively. (**D**) The expression of MAPK pathway related proteins in GPR55 low expression group and control group were detected by western-blotting in 97H cells, respectively. *N* = 3, ^***^P < 0.01, *t*-test.

The mitogen-activated protein kinase (MAPK) pathway is involved in different cellular activities in cancer development, including the transmission of extracellular signals to intracellular signals that mainly regulate cell proliferation, differentiation, migration, invasion, apoptosis, inflammation, and immunity. Abnormal activation of the MAPK pathway can lead to the occurrence of cancer. JNK, ERK, and p38 are the main subgroups of kinase-activated signaling involved in carcinogenesis, and the expression of these subgroups is often upregulated in human tumors and cancer cell lines. The MAPK pathway has been reported to be involved in the regulation of the occurrence and development of HCC. As a negative tumor regulator, we investigated the effects of *GPR55* on the stimulation of the MAPK signaling pathway. A lentivirus designed to stably overexpress *GPR55* was constructed and transfected into hepatoma cell lines (HepG2-OE and 97H-OE) and its overexpression was confirmed by western blotting. The results showed that, compared with the negative control group, after *GPR55* overexpression, both cell lines significantly inhibited the phosphorylation of key molecules in the MAPK signaling pathway (Figure 10A, 10B). We also constructed a *GPR55*-shRNA lentivirus for reverse verification. The results showed that, compared with the negative control group, the phosphorylation of ERK, JNK, and p38 in the MAPK signaling pathway was significantly enhanced after *GPR55* knockdown, indicating that *GPR55* knockdown enhanced the activation of the MAPK signaling pathway and promoted tumorigenesis (Figure 10C, 10D).

## DISCUSSION

This study evaluated the role of *GPR55* in HCC, for which there is currently limited information. The expression of *GPR55* was remarkably downregulated in HCC tissues compared to corresponding normal samples based on multiple GEO datasets. Furthermore, survival analysis revealed that high expression of *GPR55* was a protective factor for favorable overall survival and relapse-free survival in individuals with HCC. Subsequently, there was a strong relationship between *GPR55* expression and infiltration of common immune cells and immune check point proteins (CTLA4, PD-1, and PD-L1) in HCC. Importantly, high levels of *GPR55* signify better response rates to immunotherapy in HCC. In a word, our analysis demonstrated that *GPR55* is a good indicator for assessing the tumor immune microenvironment and predicting the immunotherapy responses of HCC.

*GPR55* is part of the endocannabinoid system and its biological function is still under investigation. *GPR55* is overexpressed in colon cancerous cells, and may influence ERK1/2 phosphorylation and cell proliferation [10, 11]. Carina et al. reported that *GPR55* is an oncogene in colon cancer, which could contribute to the migration and metastasis of colon cancer cells [12]. An experimental investigation revealed that *GPR55* promotes the proliferation and growth of pancreatic cancer tumors through MAPK signaling, and inhibition of *GPR55* increases the therapeutic effects of gemcitabine [13]. Furthermore, a review summarized that *GPR55* may serve as a very promising candidate for the design of new anticancer therapeutic agents [14]. However, the role of *GPR55* in HCC is still unknown. Unlike the above malignant tumors, we found that *GPR55* is downregulated in HCC tissues, and upregulation of *GPR55* is associated with a much favorable prognosis in HCC patients. More importantly, overexpression of *GPR55* by transfection in HepG2 and 97H cells significantly inhibits the growth and migration ability of HCC cells. Therefore, considering the above evidence, we may infer that *GPR55* is a tumor suppressor gene in HCC, which is in complete contrast to its role in other malignant tumors.

Survival analysis revealed that *GPR55* overexpression predicted much better survival outcomes in HCC patients, and we think that the better prognosis of *GPR55* in HCC could be partly attributed to the immune microenvironment in HCC tissues. Single-cell analysis showed that *GPR55* is expressed primarily in T cells. Next, we observed the very close relationship between *GPR55* and CD8+ T cells in HCC (r = 0.553, *P* = 9.78 × 10^−29^). CD8 +T cells are an important contributor to tumor-infiltrating lymphocytes, which play a significant role in antitumor immunity in HCC [15]. Furthermore, two recent studies have revealed that a high density of CD8+ T cells in HCC tissues correlate with fewer tumor recurrence and relatively favorable OS [16, 17]. A meta-analysis also confirmed that the presence of CD8+ T cell infiltration in HCC tissues contributes to significantly improved survival outcomes [18]. Our subsequent survival analysis grouped by CD8+ T cells demonstrated that the association between *GPR55* overexpression and better overall survival was present in the CD8 + T cell enriched group, but disappeared in the group with low CD8 + T infiltration. Therefore, our work indicates that *GPR55* influences the survival outcomes of patients with HCC in part through the regulation of T cells.

*GPR55* is widely expressed in various types of leukocytes, such as lymphocytes [7], macrophages [19], and neutrophils [20]. Previous studies have revealed that *GPR55* promotes intestinal inflammation via the infiltration of leukocytes [21]. Furthermore, flow cytometry revealed that notable levels of *GPR55* are measured in NK cells and monocytes, and modest levels can be detected in dendritic cells [8]. Our analysis through the TIMER database also demonstrated a very close relationship between *GPR55* expression and NK cells. Further, *GPR55*-mediated overstimulation of NK cells has been reported cancer, which is highly crucial in cancer immunosurveillance [21]. Our correlation analysis determined that the expression of *GPR55* was strongly related to levels of dendritic cells (r = 0.63, *P* = 5.21 × 10^−39^), CD8+ T cells (r = 0.553, *P* = 9.78 × 10^−29^), macrophages (r = 0.471, *P* = 3.47 × 10^−20^), and neutrophils (r = 0.447, *P* = 2.34 × 10^−18^), which indicates that *GPR55* could be involved in the regulation of the innate immune response. Further immunotherapy analysis demonstrated that high levels of *GPR55* were not only correlated with higher TIS, which is an objective indicator of immune response, but were also associated with better response rates to immunotherapy in HCC. Overall, *GPR55* may play a central role in the regulation of innate immune responses in HCC, representing a promising target for the immunotherapy of HCC.

Although we provided novel information on the correlation between *GPR55* and HCC, three inevitable limitations still existed in our analysis. First, we were unable to use another HCC-related data set to validate the predictive value of *GPR55* for response to immunotherapy due to technical reasons. Nonetheless, we extensively explored the association between *GPR55* expression and several immune cells in HCC based on bioinformatics, and confirmed the correlation between protein levels of *GPR55* with PD-L1 and PD-L1, but were unable to verify an association between the *GPR55* protein and immune cells. Therefore, this study still requires further confirmation through *in vitro* experiments.

## CONCLUSIONS

Our work illustrates the prognostic importance and immune correlation of *GPR55* at multiple levels via multiomic bioinformatic analysis and is supported by clinical validation. *GPR55* may influence the survival outcomes of individuals with HCC in part by regulating immune infiltration, which in turn may be vital to the regulation of immune cell infiltration and the immune response in patients with HCC.

## Supplementary Materials

Supplementary Figures

Supplementary Tables

## References

[1] Anwanwan D, Singh SK, Singh S, Saikam V, Singh R. Challenges in liver cancer and possible treatment approaches. Biochim Biophys Acta Rev Cancer. 2020; 1873:188314. 10.1016/j.bbcan.2019.188314 31682895 PMC6981221

[2] Fu J, Wang H. Precision diagnosis and treatment of liver cancer in China. Cancer Lett. 2018; 412:283–8. 10.1016/j.canlet.2017.10.008 29050983

[3] Feng GS, Hanley KL, Liang Y, Lin X. Improving the Efficacy of Liver Cancer Immunotherapy: The Power of Combined Preclinical and Clinical Studies. Hepatology. 2021 (Suppl 1); 73:104–14. 10.1002/hep.31479 32715491 PMC7854886

[4] Sangro B, Sarobe P, Hervás-Stubbs S, Melero I. Advances in immunotherapy for hepatocellular carcinoma. Nat Rev Gastroenterol Hepatol. 2021; 18:525–43. 10.1038/s41575-021-00438-0 33850328 PMC8042636

[5] Xu F, Jin T, Zhu Y, Dai C. Immune checkpoint therapy in liver cancer. J Exp Clin Cancer Res. 2018; 37:110. 10.1186/s13046-018-0777-4 29843754 PMC5975687

[6] Pierce KL, Premont RT, Lefkowitz RJ. Seven-transmembrane receptors. Nat Rev Mol Cell Biol. 2002; 3:639–50. 10.1038/nrm908 12209124

[7] Henstridge CM, Balenga NA, Kargl J, Andradas C, Brown AJ, Irving A, Sanchez C, Waldhoer M. Minireview: recent developments in the physiology and pathology of the lysophosphatidylinositol-sensitive receptor GPR55. Mol Endocrinol. 2011; 25:1835–48. 10.1210/me.2011-1197 21964594 PMC5417173

[8] Chiurchiù V, Lanuti M, De Bardi M, Battistini L, Maccarrone M. The differential characterization of GPR55 receptor in human peripheral blood reveals a distinctive expression in monocytes and NK cells and a proinflammatory role in these innate cells. Int Immunol. 2015; 27:153–60. 10.1093/intimm/dxu097 25344934

[9] Oura K, Morishita A, Tani J, Masaki T. Tumor Immune Microenvironment and Immunosuppressive Therapy in Hepatocellular Carcinoma: A Review. Int J Mol Sci. 2021; 22:5801. 10.3390/ijms22115801 34071550 PMC8198390

[10] Moon H, Ro SW. MAPK/ERK Signaling Pathway in Hepatocellular Carcinoma. Cancers (Basel). 2021; 13:3026. 10.3390/cancers13123026 34204242 PMC8234271

[11] Hasenoehrl C, Feuersinger D, Kienzl M, Schicho R. GPR55-Mediated Effects in Colon Cancer Cell Lines. Med Cannabis Cannabinoids. 2019; 2:22–8. 10.1159/000496356 34676330 PMC8489337

[12] Hasenoehrl C, Feuersinger D, Sturm EM, Bärnthaler T, Heitzer E, Graf R, Grill M, Pichler M, Beck S, Butcher L, Thomas D, Ferreirós N, Schuligoi R, et al. G protein-coupled receptor GPR55 promotes colorectal cancer and has opposing effects to cannabinoid receptor 1. Int J Cancer. 2018; 142:121–32. 10.1002/ijc.31030 28875496 PMC5679368

[13] Ferro R, Adamska A, Lattanzio R, Mavrommati I, Edling CE, Arifin SA, Fyffe CA, Sala G, Sacchetto L, Chiorino G, De Laurenzi V, Piantelli M, Sansom OJ, et al. GPR55 signalling promotes proliferation of pancreatic cancer cells and tumour growth in mice, and its inhibition increases effects of gemcitabine. Oncogene. 2018; 37:6368–82. 10.1038/s41388-018-0390-1 30061636

[14] Leyva-Illades D, Demorrow S. Orphan G protein receptor GPR55 as an emerging target in cancer therapy and management. Cancer Manag Res. 2013; 5:147–55. 10.2147/CMAR.S35175 23869178 PMC3706254

[15] Huang CY, Wang Y, Luo GY, Han F, Li YQ, Zhou ZG, Xu GL. Relationship Between PD-L1 Expression and CD8+ T-cell Immune Responses in Hepatocellular Carcinoma. J Immunother. 2017; 40:323–33. 10.1097/CJI.0000000000000187 29028787

[16] Ramzan M, Sturm N, Decaens T, Bioulac-Sage P, Bancel B, Merle P, Tran Van Nhieu J, Slama R, Letoublon C, Zarski JP, Jouvin-Marche E, Marche PN, Leroy V. Liver-infiltrating CD8(+) lymphocytes as prognostic factor for tumour recurrence in hepatitis C virus-related hepatocellular carcinoma. Liver Int. 2016; 36:434–44. 10.1111/liv.12927 26215124

[17] Guo M, Yuan F, Qi F, Sun J, Rao Q, Zhao Z, Huang P, Fang T, Yang B, Xia J. Expression and clinical significance of LAG-3, FGL1, PD-L1 and CD8(+)T cells in hepatocellular carcinoma using multiplex quantitative analysis. J Transl Med. 2020; 18:306. 10.1186/s12967-020-02469-8 32762721 PMC7409704

[18] Ding W, Xu X, Qian Y, Xue W, Wang Y, Du J, Jin L, Tan Y. Prognostic value of tumor-infiltrating lymphocytes in hepatocellular carcinoma: A meta-analysis. Medicine (Baltimore). 2018; 97:e13301. 10.1097/MD.0000000000013301 30557978 PMC6320107

[19] Stančić A, Jandl K, Hasenöhrl C, Reichmann F, Marsche G, Schuligoi R, Heinemann A, Storr M, Schicho R. The GPR55 antagonist CID16020046 protects against intestinal inflammation. Neurogastroenterol Motil. 2015; 27:1432–45. 10.1111/nmo.12639 26227635 PMC4587547

[20] Balenga NA, Aflaki E, Kargl J, Platzer W, Schröder R, Blättermann S, Kostenis E, Brown AJ, Heinemann A, Waldhoer M. GPR55 regulates cannabinoid 2 receptor-mediated responses in human neutrophils. Cell Res. 2011; 21:1452–69. 10.1038/cr.2011.60 21467997 PMC3132458

[21] Waldhauer I, Steinle A. NK cells and cancer immunosurveillance. Oncogene. 2008; 27:5932–43. 10.1038/onc.2008.267 18836474

