# Supplementary material for: A comprehensive survival and prognosis analysis of GPR55 expression in hepatocellular carcinoma
Source: Aging (Albany NY). 2023 Sep 8;15(17):8930–47. doi: 10.18632/aging.205008 (PMC10522392; doi:10.18632/aging.205008)
Supplement: Supplementary Figures [file aging-15-205008-s001.pdf]

SUPPLEMENTARY FIGURES

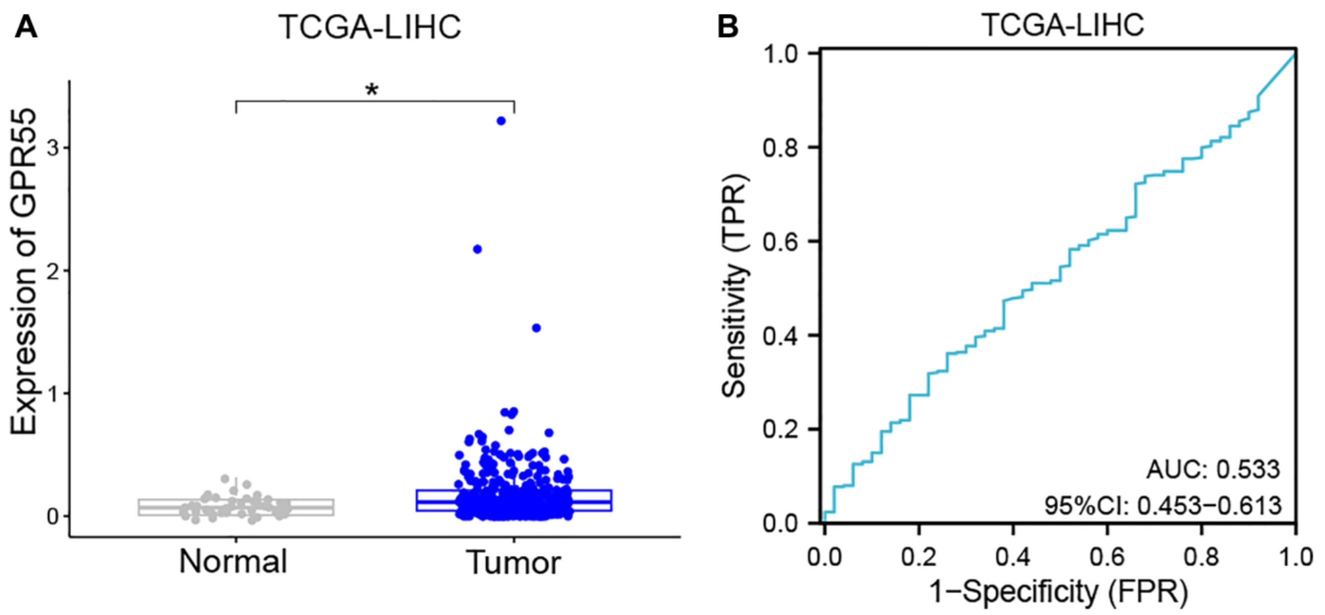

Supplementary Figure 1. (**A**) Comparison of GPR55 expression in normal and tumor tissues in TCGA-LIHC dataset (*$P < 0.05$). (**B**) ROC diagnostic curve of GPR55 in TCGA-LIHC dataset.

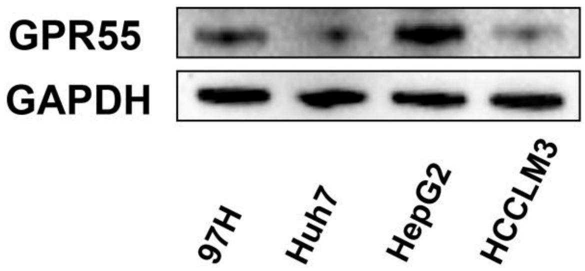

Supplementary Figure 2. Protein levels of GPR55 were detected by western-blotting in different hepatocellular carcinoma cell lines.
