# Supplementary material for: A comprehensive survival and prognosis analysis of GPR55 expression in hepatocellular carcinoma
Source: Aging (Albany NY). 2023 Sep 8;15(17):8930–47. doi: 10.18632/aging.205008 (PMC10522392; doi:10.18632/aging.205008)
Supplement: Supplementary Tables [file aging-15-205008-s002.pdf]

SUPPLEMENTARY TABLES

Supplementary Table 1. Survival analysis of GPR55 in different malignant tumors.

Cancer type	HR	Lower 95% CI	Upper 95% CI	*P* value	HR	Lower 95% CI	Upper 95% CI	*P* value
BLCA	1.11	0.82	1.49	0.5	0.64	0.28	1.5	0.3
BRCA	**0.58**	**0.4**	**0.83**	**0.025**	**0.65**	**0.42**	**1**	**0.046**
CESC	0.56	0.33	0.95	0.031	0.4	0.18	0.86	0.016
ESCA	3.5	1.35	9.1	0.007	0.59	0.22	1.59	0.29
ESCA	0.64	0.34	1.21	0.17	2.73	0.28	26.42	0.37
HNSC	**0.57**	**0.44**	**0.75**	**5.3*e-5**	**2.53**	**1.19**	**5.37**	**0.012**
KIRC	0.83	0.6	1.14	0.24	2.35	0.84	6.57	0.093
KIRP	1.81	0.97	3.37	0.059	2.98	1.24	7.17	0.011
LIHC	**0.66**	**0.47**	**0.93**	**0.018**	**0.51**	**0.36**	**0.71**	**7.2*e-5**
LUAD	0.62	0.46	0.83	0.0013	0.69	0.45	1.05	0.083
LUSC	1.21	0.91	1.61	0.18	1.37	0.8	2.37	0.25
OV	1.14	0.87	1.49	0.35	0.61	0.42	0.89	0.0091
PAAD	0.68	0.45	1.03	0.07	0.68	0.29	1.58	0.37
PCPG	0.32	0.06	1.84	0.18	2.2	0.3	16.35	0.43
READ	1.72	0.76	3.86	0.19	0.33	0.07	1.64	0.15
SARC	0.57	0.37	0.87	0.0077	0.68	0.38	1.21	0.19
STAD	0.8	0.57	1.13	0.21	0.64	0.33	1.27	0.2
TGCT	5.6	0.51	61.79	0.11	0.25	0.06	1.07	0.044
THCA	0.17	0.02	1.29	0.052	0.73	0.33	1.6	0.42
UCEC	**0.53**	**0.35**	**0.8**	**0.0024**	**0.45**	**0.27**	**0.75**	**0.0018**

Supplementary Table 2. Analysis of the correlation between the level of GPR55 and the infiltration of immune cells in HCC.

Cell type	Gene markers	None		Purity	
		Corr	*P* value	Corr	*P* value
**T cell**	CD2	0.712	1.26*e-58	0.648	1.96*e-42
	CD3D	0.616	4.3*e-40	0.537	3.35*e-27
	CD3E	0.735	3.17*e-64	0.676	1.95*e-47
**CD8+ T cell**	CD8A	0.646	3.39*e-45	0.577	5.26*e-32
	CD8B	0.58	1.09*e-34	0.501	2.41*e-23
**B cell**	CD19	0.521	3.75*e-27	0.434	2.58*e-17
	CD79A	0.637	1.39*e-43	0.548	1.99*e-28
**TAM**	IL10	0.54	1.76*e-29	0.415	9.03*e-16
	CD68	0.452	4.7*e-20	0.323	8.51*e-10
	CCL2	0.518	7.16*e-27	0.379	3.34*e-13
**M1 cell**	NOS2	0.064	0.222	0.028	0.6
	IRF5	0.253	8.11*e-7	0.269	4.02*e-7
	PTGS2	0.515	1.66*e-26	0.395	2.39*e-14
**M2 cell**	MS4A4A	0.582	4.81*e-35	0.481	2.1*e-21
	VSIG4	0.517	9.95*e-27	0.399	1.38*e-4
	CD163	0.581	8.26*e-35	0.475	7.3*e-21
**Monocyte**	CSF1R	0.693	263*e-54	0.609	2.3*e-36
	CD86	0.694	1.19*e-54	0.622	2.93*e-38

**Dendritic cell**	CD1C	0.693	1.96*e-54	0.641	2.29*e-41
	NRP1	0.395	2.72*e-15	0.364	2.97*e-12
	ITGAX	0.617	2.63*e-40	0.535	5.44*e-27
**Neutrophil**	CCR7	0.652	2.77*e-46	0.559	1.09*e-29
	ITGAM	0.438	7.55*e-19	0.349	2.61*e-11
	CEACAM8	0.095	0.00679	0.068	0.209
**NK cell**	KIR3DL1	0.218	2.33*e-5	0.19	3.95*e-4
	KIR3DL2	0.35	3.95*e-12	0.296	2.08*e-8
	KIR3DL3	0.171	9.54*e-4	0.16	2.8*e-3
	KIR2DS4	0.267	1.88*e-7	0.284	8.32*e-8
	KIR2DL1	0.159	0.00208	0.125	0.0205
	KIR2DL3	0.306	1.75*e-9	0.266	5.44*e-7
	KIR2DL4	0.392	4.52*e-15	0.36	5.78*e-12
